# *Gata4* Is Required for Formation of the Genital Ridge in Mice

**DOI:** 10.1371/journal.pgen.1003629

**Published:** 2013-07-11

**Authors:** Yueh-Chiang Hu, Leah M. Okumura, David C. Page

**Affiliations:** Whitehead Institute, Howard Hughes Medical Institute, and Department of Biology, Massachusetts Institute of Technology, Cambridge, Massachusetts, United States of America; Stowers Institute for Medical Research, United States of America

## Abstract

In mammals, both testis and ovary arise from a sexually undifferentiated precursor, the genital ridge, which first appears during mid-gestation as a thickening of the coelomic epithelium on the ventromedial surface of the mesonephros. At least four genes (*Lhx9*, *Sf1*, *Wt1*, and *Emx2*) have been demonstrated to be required for subsequent growth and maintenance of the genital ridge. However, no gene has been shown to be required for the initial thickening of the coelomic epithelium during genital ridge formation. We report that the transcription factor GATA4 is expressed in the coelomic epithelium of the genital ridge, progressing in an anterior-to-posterior (A-P) direction, immediately preceding an A-P wave of epithelial thickening. Mouse embryos conditionally deficient in *Gata4* show no signs of gonadal initiation, as their coelomic epithelium remains a morphologically undifferentiated monolayer. The failure of genital ridge formation in *Gata4*-deficient embryos is corroborated by the absence of the early gonadal markers LHX9 and SF1. Our data indicate that GATA4 is required to initiate formation of the genital ridge in both XX and XY fetuses, prior to its previously reported role in testicular differentiation of the XY gonad.

## Author Summary

During mammalian fetal development, the precursor of the testis or ovary first appears as a simple thickening, in a specific region, of the epithelial cell layer that lines the body cavity. The resulting structure is called the genital ridge, which then differentiates into either testis or ovary, depending on whether the sex chromosome constitution is XY or XX. A handful of genes, including *Lhx9*, *Sf1*, *Wt1*, and *Emx2*, are required to sustain the growth of the genital ridge. However, mice with mutations in any of these genes still undergo the initial step of epithelial thickening, suggesting that an additional step (or factor) is required to initiate genital ridge formation. We found that the evolutionarily conserved transcription factor GATA4 is expressed in the epithelium of the genital ridge before initial thickening. We produced a mouse with a *Gata4* mutation in this tissue and demonstrated that the initial thickening does not take place; the mutant embryos fail to initiate gonad development. In support of this observation, the *Gata4* mutant does not express the early gonadal markers LHX9 and SF1. These findings indicate that a genetically discrete, *Gata4*-dependent initiation step precedes the previously known processes that result in formation of testes and ovaries.

## Introduction

In male and female mammals alike, the embryonic gonad initially forms as a sexually bipotential structure called the genital ridge. The genital ridge subsequently differentiates to become a testis or an ovary.

Formation of the genital ridge begins with increasing proliferation of coelomic epithelial cells to establish a dense and pseudostratified layer on the ventromedial surface of the mesonephros [1]–[4]. At about the same time, the underlying basement membrane becomes fragmented, allowing the epithelial cells to migrate inward and form a thickened, multilayered structure [5]–[7]. These morphological changes, which first appear anteriorly and gradually extend in a posterior direction, create the genital ridge [1], [3], [4], [8].

In mouse embryos, formation of the genital ridge starts at about embryonic day 10.5 (E10.5) and continues until E11.5-E12.0, when sexual differentiation of the gonad becomes evident [9], [10]. Full development of the genital ridge requires a set of genes that includes *Steroidogenic factor 1* (*Sf1*) [11], *Lim homeobox protein 9* (*Lhx9*) [12], *Wilms tumor 1* (*Wt1*) [13], and *Empty spiracles homeobox 2* (*Emx2*) [7], [14]. In mouse embryos homozygous for a null mutation in any one of these genes, the coelomic epithelial layer shows initial thickening but regresses before the genital ridge is fully formed. Deficiency of either *Sf1* or *Wt1* results in the death of somatic cells within the developing genital ridge, whereas loss of *Lhx9* disrupts proliferation of these cells [11], [12], [15], [16]. *Emx2* deletion impairs the migration of epithelial cells through the basement membrane to form a multilayered structure [7]. Each of these genes is thus required for growth and maintenance of the genital ridge, but not for its initial formation.

How the coelomic epithelium begins to differentiate into the genital ridge remains unknown. Previously reported mouse mutants that lack gonads during fetal development show signs of epithelial thickening or various levels of gonadal development before its regression. Examples include those mentioned above and *Osr1*-null embryos [17] (Y.C. Hu and D.C. Page, unpublished data). Mouse mutants lacking the epithelial thickening that heralds the genital ridge have not yet been reported.

GATA4 is an evolutionarily conserved transcription factor that is essential for early development of multiple organs, including heart, foregut, liver, and ventral pancreas [18]–[20]. Interestingly, *Gata4* is also expressed in the genital ridge, and this expression pattern is conserved across many organisms, including mammals [21]–[23], chicken [24], fish [25], and turtles [26]. *Gata4*'s expression in the genital ridge has been linked to its role in testis differentiation. Specifically, GATA4, together with WT1, synergistically activates transcription of *Sry* [27], which triggers testicular differentiation of the genital ridge. In XY mouse embryos homozygous for a *Gata4* knock-in allele (*Gata4^ki^*) that abrogates GATA4 binding to the cofactor FOG2 (or FOG1), genital ridges form, but further differentiation into testes is blocked, and the transcriptional program downstream of *Sry* is greatly attenuated [28]. Mouse embryos heterozygous for *Gata4^ki^* on specific genetic backgrounds also show sex reversal of genetic males to phenotypic females [29]. In another study where *Gata4* was removed conditionally from XY genital ridges after E10.5, subsequent testis differentiation was disrupted and ovarian somatic markers were upregulated [30]. These studies clearly indicate a requirement for *Gata4* in testis determination and differentiation.

In this study, we investigated whether *Gata4* plays a role in formation of the genital ridge, prior to the point of testis determination. *Gata4*-null embryos die before the genital ridges form [19], [20], so we utilized the tamoxifen-inducible Cre/loxP system to conditionally delete *Gata4* in mouse embryos after E8.75. Our approach allows mutant embryos to survive to ∼E11.5, thus providing us an opportunity to investigate *Gata4*'s role in early gonadal development. Here we report that embryos conditionally deficient for *Gata4* show neither coelomic epithelial thickening nor expression of the early gonadal differentiation factors LHX9 and SF1. Therefore, our study indicates that *Gata4* is required for formation of the genital ridge, prior to its previously reported role in testis determination.

## Results

### GATA4 expression precedes thickening of genital ridge epithelium and progresses in an A-P direction

The earliest stage of gonadogenesis is characterized by epithelial thickening, which begins in mouse embryos at ∼E10.5 (8 tail somite [ts] stage) [5], [7]. According to morphological studies, formation of the genital ridge progresses in an A-P direction along the axis of the mesonephros [1]. If GATA4 is involved in the signaling pathway that drives genital ridge formation, it should be expressed in the coelomic epithelium before initial thickening and ideally in a similar A-P direction. In order to investigate the timing and location of GATA4 expression, we performed whole-mount immunofluorescence on C57BL/6 embryos at E10.0, E10.2 and E10.4.

**Figure 1:**
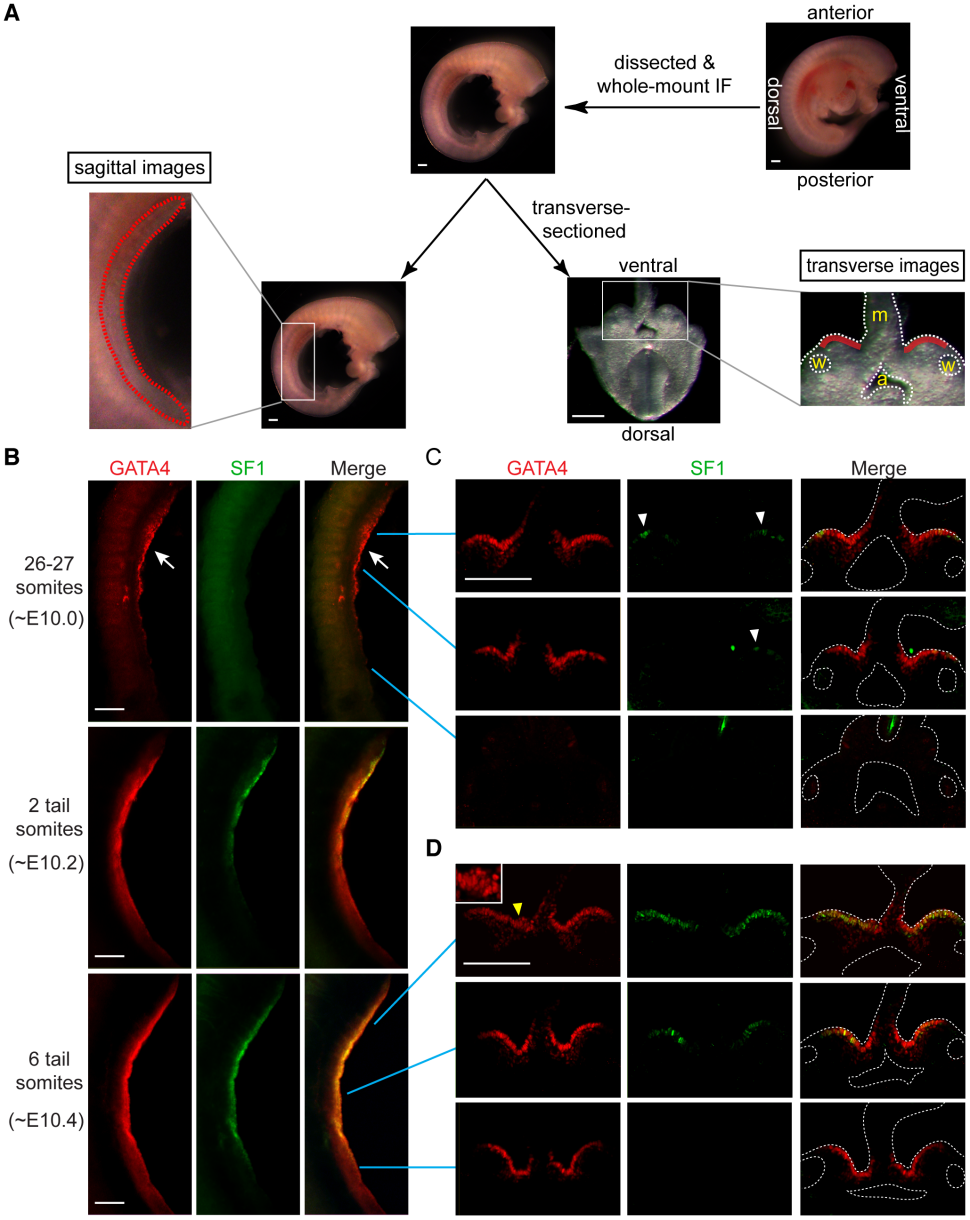
GATA4 expression precedes coelomic epithelial thickening and progresses from anterior to posterior. (A) Schematic representation of experiment. Mouse embryos were dissected to remove limbs, body walls and internal organs, and then subjected to whole-mount immunofluorescence (IF) staining with GATA4 and SF1 antibodies. Stained embryos were imaged sagittally by confocal microscopy, and then transversely (following transverse section), again by confocal microscopy. Red dashed and solid lines in, respectively, sagittal and transverse images indicate location of developing gonads. a, dorsal aorta; m, mesentery; w, Wolffian duct. (B–D) Expression analysis of GATA4 (red) and SF1 (green) protein during early gonadogenesis. GATA4 expression in coelomic epithelia of genital ridges begins in anterior (arrow) and then spreads posteriorly. Epithelial thickening is observed in anterior region of genital ridge at 6-tail-somite stage (yellow arrowhead and inset). SF1 (white arrowheads) is expressed only sporadically in anterior half at 26–27 somite stage. Scale bars: 50 µm.

The nascent gonads first appear as a paired coelomic epithelial layer lying along the surface of the mesonephroi, lateral to the dorsal mesentery, and extending between the front and hind limbs. After dissection to expose the entire length of the genital ridges, we were able to image them longitudinally by confocal laser scanning microscopy (Figure 1A). We found that GATA4 protein was present in the anterior half of the genital ridge as early as ∼E10.0 (26–27 total somites) and extended to the posterior half by ∼E10.2 (2 ts) (Figure 1B). Thus, expression of GATA4 protein progressed in an A-P direction. This expression did not extend into the metanephric region (Figure S1), suggesting that GATA4 is restricted to the region that will later form the genital ridge.

*Sf1* has been suggested as one of the earliest markers of gonadal cells [31]. Therefore, we compared the expression of GATA4 and SF1 proteins in the same tissues. In the sagittal images, SF1 was clearly detected at the anterior half of the genital ridge at ∼E10.2 (2 ts) and later extended into the posterior half (Figure 1B). Similar to GATA4, SF1 was also expressed in an A-P direction. However, expression of GATA4 preceded expression of SF1.

To confirm and extend these observations, we transversely sectioned the stained embryos with a vibratome and took sections from anterior, middle, and posterior parts of the genital ridges for confocal imaging (Figure 1A). Consistent with the results from the sagittal images, at ∼E10.0 GATA4 was expressed homogeneously in the coelomic epithelial layer of the genital ridge from the anterior to the middle portions, while SF1 was expressed only sporadically in the same areas (Figure 1C, white arrowheads). At ∼E10.4 (6 ts), GATA4 expression had already spread to the posterior end of the genital ridge, while SF1 expression had spread only to the middle region (Figure 1D). Therefore, the A-P expression of GATA4 was earlier than that of SF1, suggesting the possibility that GATA4 might function upstream of SF1. In addition, we consistently observed that the coelomic epithelium at the anterior of the ridge was developmentally more advanced than that at the posterior. For instance, at ∼E10.4, the coelomic epithelium already showed more than one layer of cells in the anterior region (Figure 1D, yellow arrowhead), while it remained a monolayer in the middle-to-posterior areas. Taken together, these findings demonstrate that GATA4 is expressed in the genital ridge epithelium in an A-P direction and just before thickening occurs, thus fitting the profile of a candidate gene that regulates the formation of the genital ridge.

### *Gata4* is required for genital ridge formation

Having observed that expression of GATA4 correlates with genital ridge formation, we wanted to know whether GATA4 is required for the earliest event, the initial thickening of the coelomic epithelium. *Gata4*-null embryos die between E7.5 and E9.5 [19], [20], so we used the tamoxifen-inducible Cre/loxP system to inactivate *Gata4* gene activity in a temporally specific manner. We first intercrossed *CAG-CreER*; *Gata4^+/Δ^* male to *Gata4^flox/flox^* female mice to produce embryos carrying *CAG-CreER*; *Gata4^flox/Δ^*, where the *CAG* promoter drives ubiquitous expression of *CreER* [32]. We then injected the dams with tamoxifen at E8.75 days to generate *Gata4* conditional knockout embryos, hereafter referred to as *Gata4* cKO (*CAG-CreER*). *Gata4^flox/+^* embryos from the same litters served as controls.

**Figure 2:**
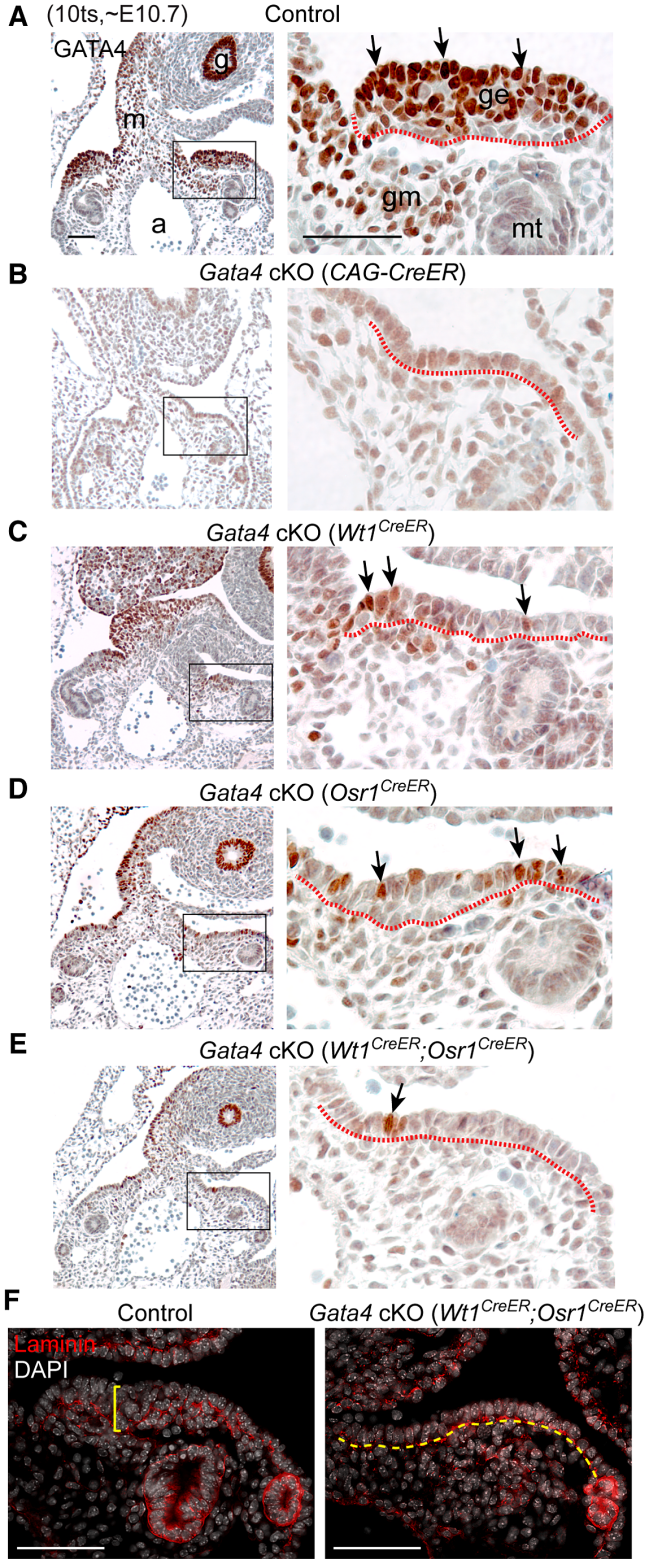
*Gata4* is required for thickening of coelomic epithelium that gives rise to genital ridge. (A–E) IHC staining for GATA4 protein in transverse sections of control and *Gata4* cKO embryos. Sections were chosen to represent similar anterior positions in genital ridges. Right panel shows higher magnification of boxed area in left panel. Arrows indicate examples of positive GATA4 staining. Red dashed lines mark boundary between coelomic epithelium and underlying mesenchyme. Control and conditional mutant embryos, except for *Gata4* cKO (*CAG-CreER*), were from the same litter. a, dorsal aorta; g, gut endoderm; ge, genital ridge epithelium; gm, genital mesenchyme; m, mesentery; mt, mesonephric tubule. (F) Immunofluorescent staining for laminin protein in sections of control and *Gata4* cKO embryos. Laminin marks basement membrane. Control genital ridge shows thickened epithelial layer (yellow bracket), whereas the coelomic epithelium in *Rb* cKO embryo remains a single-cell layer (yellow dashed line). Scale bars: 50 µm.

We first examined whether GATA4 expression was ablated by our conditional deletion strategy, and whether the epithelial thickening was affected in mutant embryos. Following tamoxifen treatment, *Gata4* cKO (*CAG-CreER*) embryos died between E11.0 and E11.5, likely due to the requirement for *Gata4* in early development of multiple organs, including the heart, gut, and liver. We therefore collected embryos at ∼E10.7 (10 ts). In control embryos, GATA4 was clearly detected in the thickened coelomic epithelium of the developing gonad (Figure 2A, arrows) as well as in cells of the genital mesenchyme, mesentery, and gut endoderm. In *Gata4* cKO (*CAG-CreER*) embryos, GATA4 was almost undetectable in the corresponding coelomic epithelium and in the rest of the embryo section, indicating that the tamoxifen-induced *Gata4* deletion was nearly complete (Figure 2B). We found that the coelomic epithelium of mutant embryos remained as a single layer of cells (Figure 2B), suggesting that initial thickening was disrupted by the loss of *Gata4*.

Given that *Gata4* has a widespread function in organogenesis, we wanted to confirm that the lack of epithelial thickening is a direct consequence of *Gata4* removal in the genital ridges instead of a secondary effect caused by ubiquitous deletion of *Gata4* via *CAG-CreER*. We used two tissue-specific CreER systems, *Osr1-eGFP-CreERt2* [33] and *Wt1-CreERt2* [34], to replace the *CAG-CreER* in the experiment described above. In these two systems, *eGFP-CreERt2* and *CreERt2* cassettes were targeted into the endogenous *Osr1* and *Wt1* loci, respectively, to create knock-in alleles. *Osr1* is expressed in the intermediate mesoderm and its derivatives, including genital ridges, with peak expression between E8.5 and E9.5 [35]. The gene *Wt1* is thought to act downstream of *Osr1* and is detected in the urogenital ridges starting at ∼E9.5 [17], [36]. *Wt1* and *Osr1* are also expressed in the embryonic heart and some other tissues during the time frame of the study. The temporal and spatial activity of the *CreERt2* cassettes in *Osr1^eGFP-CreERt2/+^* and *Wt1^CreERt2/+^* embryos correlated well with that of the respective endogenous genes, as evaluated by the CAG-LSL-tdTomato reporter using *Gt(ROSA)26Sor^CAG-tdTomato^* mice (data not shown). We then introduced these knock-in alleles into *Gata4^+/Δ^* males, which were bred with *Gata4^flox/flox^* females to produce *Gata4* conditional mutant embryos. *Gata4* deletion efficiency in the genital ridge was assayed by immunohistochemical staining for GATA4 protein.

Following the previously described tamoxifen injection scheme, we assessed GATA4 expression and gonadal phenotype of the mutant embryos carrying *Wt1^CreERt2/+^*; *Gata4^flox/Δ^* or *Osr1^eGFP-CreERt2/+^*; *Gata4^flox/Δ^*, hereafter referred to as *Gata4* cKO (*Wt1^CreER^*) and *Gata4* cKO (*Osr1^CreER^*), respectively. Compared to the controls at E10.7 (10 ts), the number of GATA4-expressing cells was greatly reduced in the mutant coelomic epithelium, while GATA4 expression in the mesentery and gut endoderm remained unaffected (Figure 2C, 2D). Thickening of the coelomic epithelium was much less prominent in both mutants (Figure 2C, 2D). To improve the efficiency of tamoxifen-induced *Gata4* deletion, we introduced both knock-in alleles into the same animals, hereafter referred to as *Gata4* cKO (*Wt1^CreER^*;*Osr1^CreER^*). As expected, we obtained a greater degree of reduction in the number of GATA4-expressing cells in the coelomic epithelium of *Gata4* cKO (*Wt1^CreER^*;*Osr1^CreER^*) embryos, and the coelomic epithelium remained as a single cell layer of cells (Figure 2E). However, it should be noted that the efficiency of *Wt1^CreER^*- and/or *Osr1^CreER^*-mediated *Gata4* deletion varied among mutants. Only mutants that displayed a high degree of *Gata4* deletion were chosen for detailed study, and these embryos died between E11.0 and E11.5. Mutant embryos that survived beyond E12.0 did not show sufficient deletion of *Gata4* to block gonadal initiation.

To assess the degree of epithelial thickening in control and mutant embryos, we determined the number of cell layers by staining for the basement membrane with an antibody against laminin. While control genital ridges had grown to a multilayered epithelial structure, the mutant coelomic epithelium remained a single cell layer without clear signs of thickening (Figure 2F). Thus, we conclude that *Gata4* is essential for the initial thickening of the coelomic epithelium that gives rise to the genital ridge. Note that the absence of this thickening was seen in both XX and XY *Gata4* cKO mutants, which is consistent with the fact that genital ridge formation precedes gonadal sex differentiation.

### *Gata4* deficiency impairs epithelial proliferation and basement membrane fragmentation

**Figure 3:**
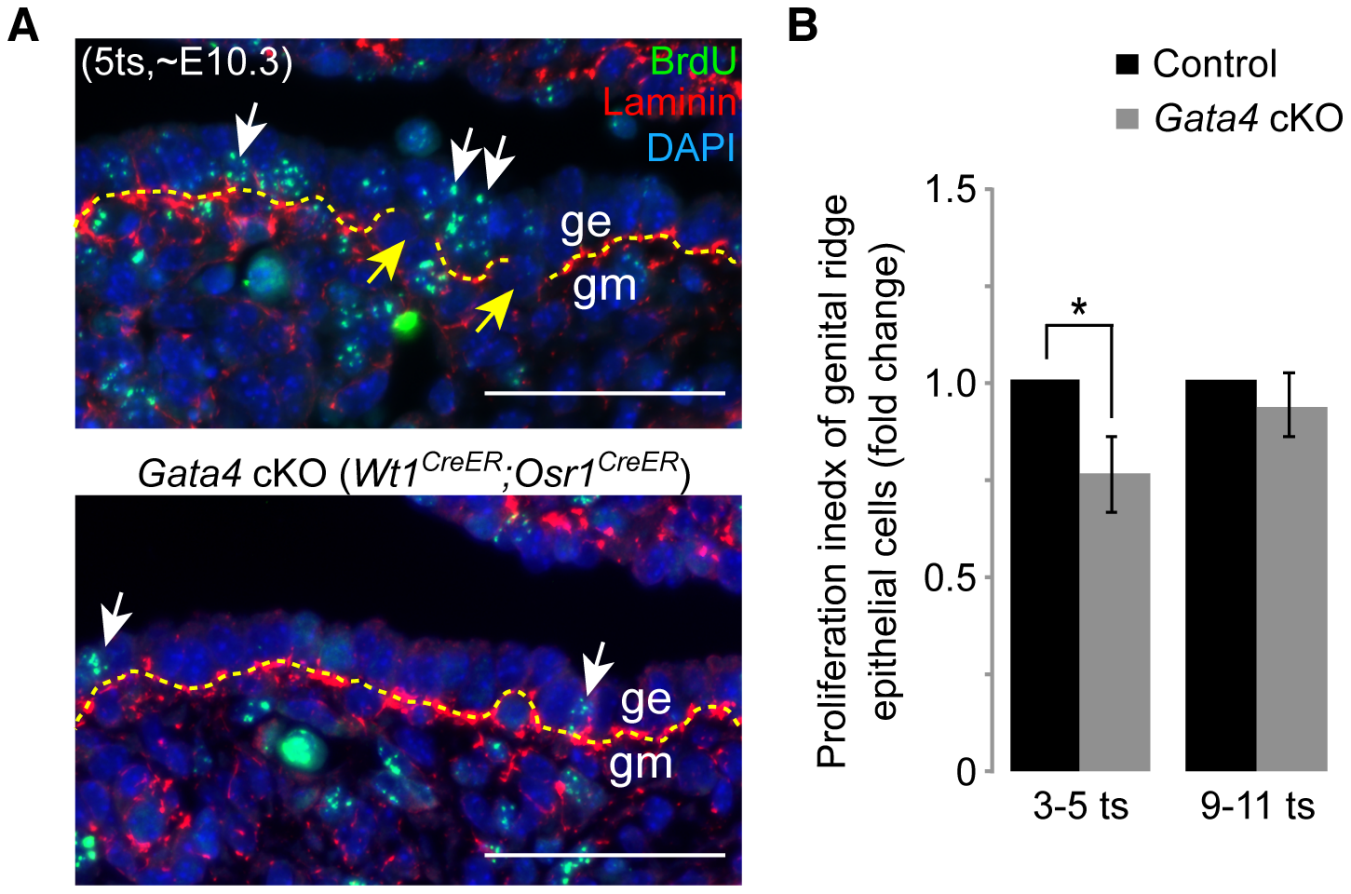
*Gata4* deficiency impairs epithelial proliferation and basement membrane breakdown. (A) Immunofluorescent staining for BrdU (green) and laminin (red) in sections of control and *Gata4* cKO (*Wt1^CreER^*;*Osr1^CreER^*) embryos where tamoxifen and BrdU were injected at, respectively, E8.75 and ∼E10.0 (6 hours before sacrifice). Yellow dashed lines mark basement membrane. Yellow arrows mark discontinuities in basement membrane in control genital ridge. White arrows indicate representative BrdU-positive epithelial cells. Nuclei stained with DAPI (blue). ge, genital ridge epithelium; gm, genital mesenchyme. Scale bars: 50 µm. (B) Relative proliferation index, comparing the fractions of coelomic epithelial cells positive for BrdU in control and *Gata4* cKO (*Wt1^CreER^*;*Osr1^CreER^*) embryos of the same sex, from the same litter. The index in controls was set at 1. Germ cells were excluded from the counting. At each of the two stages shown, three pairs of control and *Gata4* cKO embryos were studied. Plotted here are means ± standard deviation. *, *P*<0.05 (two-tailed Student's *t*-test).

Epithelial thickening is first observed around E10.3–E10.4 (5–6 ts) at the anterior genital ridge. It has been reported that an increase in proliferation of the coelomic epithelium immediately precedes the thickening event [1], [3], [4], [37]. Therefore, we tested whether the lack of epithelial thickening in *Gata4* cKO embryos is caused by lack of epithelial cell proliferation. We collected BrdU-treated control and *Gata4* cKO (*Wt1^CreER^*;*Osr1^CreER^*) embryos at two different time points: ∼E10.3 (3–5 ts) and ∼E10.7 (9–11 ts), representing just before and after the occurrence of epithelial thickening. After immunostaining sections with antibodies against BrdU and laminin (Figure 3A), we analyzed the fraction of gonadal somatic cells that had incorporated BrdU. We found that the control epithelium exhibited a significantly higher proliferation rate, compared to the *Gata4* cKO (*Wt1^CreER^*;*Osr1^CreER^*) epithelium, just before thickening occurred (Figure 3B). After thickening had taken place, the control epithelium proliferated at a similar rate as the mutant epithelium (Figure 3B). These data suggest that *Gata4* is required to increase the proliferation of the coelomic epithelium, which seemingly contributes to the subsequent thickening event.

For thickening to occur, the basement membrane underneath the coelomic epithelium has to lose its continuity, permitting epithelial cells to migrate inward to form additional layers. To determine the integrity of the basement membrane underneath control and *Gata4* cKO (*Wt1^CreER^*;*Osr1^CreER^*) coelomic epithelia, we labeled embryo sections with laminin antibody. We observed that the basement membrane in the controls had become fragmented by ∼E10.3 (5 ts) (Figure 3A, yellow arrows). In contrast, the basement membrane in the mutants remained continuous, thus hindering the thickening process. Therefore, *Gata4* cKO coelomic epithelium does not show any features of gonadal differentiation.

To determine whether increased cell death also contributed to the lack of epithelial thickening in *Gata4* cKO embryos, we measured cell apoptosis by TUNEL staining. We found that mutant coelomic epithelial layers showed slightly higher TUNEL labeling indices compared to controls, but the difference did not reach statistical significance (data not shown).

### Expression of LHX9 and SF1 is dependent on *Gata4*

To substantiate the finding that *Gata4* deficiency disrupts formation of the genital ridge, we determined whether expression of early gonadal regulators was affected in *Gata4* cKO mutants. Each of the genes *Lhx9*, *Sf1*, *Wt1*, and *Emx2* is known to control the growth and maintenance of the genital ridge [11]–[14]. *Lhx9* and *Sf1* are expressed specifically in the coelomic epithelium of the developing genital ridge, whereas *Wt1* and *Emx2* are expressed more broadly. Having found that GATA4 is primarily expressed in the coelomic epithelial layer of the genital ridge during the initiation period (Figure 1), we first tested whether LHX9 and SF1 are regulated by *Gata4*, using whole-mount immunofluorescence. We examined embryos with near complete deletion of *Gata4* at ∼E10.7–E10.8 for the experiment.

**Figure 4:**
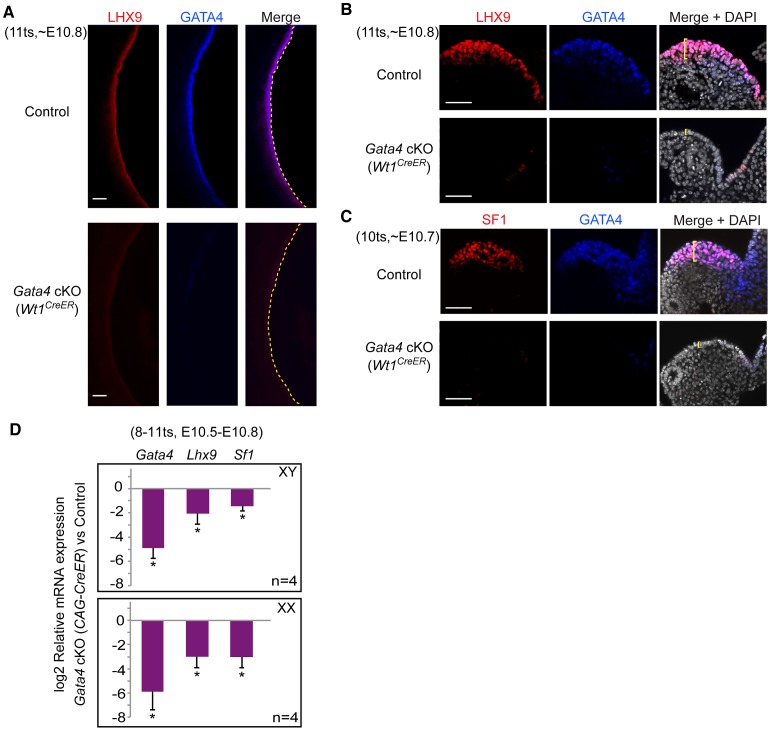
*Gata4* is required for expression of early gonadal differentiation regulators LHX9 and SF1. (A–C) Whole-mount immunofluorescent staining for LHX9, SF1, and GATA4 protein in control and *Gata4* cKO (*Wt1^CreER^*) embryos. Confocal images were taken either sagittally (A) or transversely (B and C). Yellow dashed lines outline coelomic epithelial surface. Yellow brackets indicate thickness of epithelial layer. Scale bars: 50 µm. (D) Quantitative analysis of *Gata4*, *Lhx9*, and *Sf1* mRNA levels in control and *Gata4* cKO (*CAG-CreER*) urogenital ridges. Plotted here are means ± standard deviation from biological replicates (all values normalized to *beta-actin*). *, *P*<0.001 (two-tailed Student's *t*-test).

In control embryos, both GATA4 and LHX9 were co-expressed and co-localized in the epithelial cells along the length of the genital ridges (Figure 4A, 4B). By contrast, the *Gata4* cKO (*Wt1^CreER^*) coelomic epithelium showed minimal expression of GATA4 and LHX9 (Figure 4A, 4B). There was some residual expression at the epithelium of the dorsal mesentery. Likewise, in control genital ridges, SF1 was restricted to the thickened epithelial layer, and all SF1-positive cells were also GATA4-positive (Figure 4C). SF1 did not extend to the dorsal mesentery, where many cells expressed GATA4 alone. By contrast, SF1 was absent in the coelomic epithelium of both *Gata4* cKO (*Wt1^CreER^*) and *Gata4* cKO (*CAG-CreER*) embryos (Figure 4C and Figure S2). These results suggest that *Gata4* is required for expression of LHX9 and SF1, and that these two factors act downstream of GATA4. Notably, *Gata4* cKO mutants that failed to express LHX9 or SF1 also did not exhibit thickening of the coelomic epithelium (Figure 4B, 4C, yellow brackets).

To further confirm the loss of *Lhx9* and *Sf1* expression in the absence of *Gata4*, we measured the mRNA expression levels of these genes in XY and XX urogenital ridges that contained gonad/mesonephros complexes and the dorsal part of the mesentery. In this experiment, we used *Gata4* cKO embryos carrying *CAG-CreER*, instead of *Wt1^CreER^* or *Osr1^CreER^*, to avoid any potential influence of *Osr1* or *Wt1* haploinsufficiency on gonadal gene expression. As expected, at E10.5–E10.8 (8–11 ts), mRNA for *Lhx9* and *Sf1* was significantly reduced in the *Gata4* cKO samples of both sexes (Figure 4D). These findings indicate that *Gata4* cKO coelomic epithelial cells fail to express the early gonadal differentiation regulators *Lhx9* and *Sf1*, supporting the hypothesis that *Gata4* is required for initiation of genital ridge formation.

**Figure 5:**
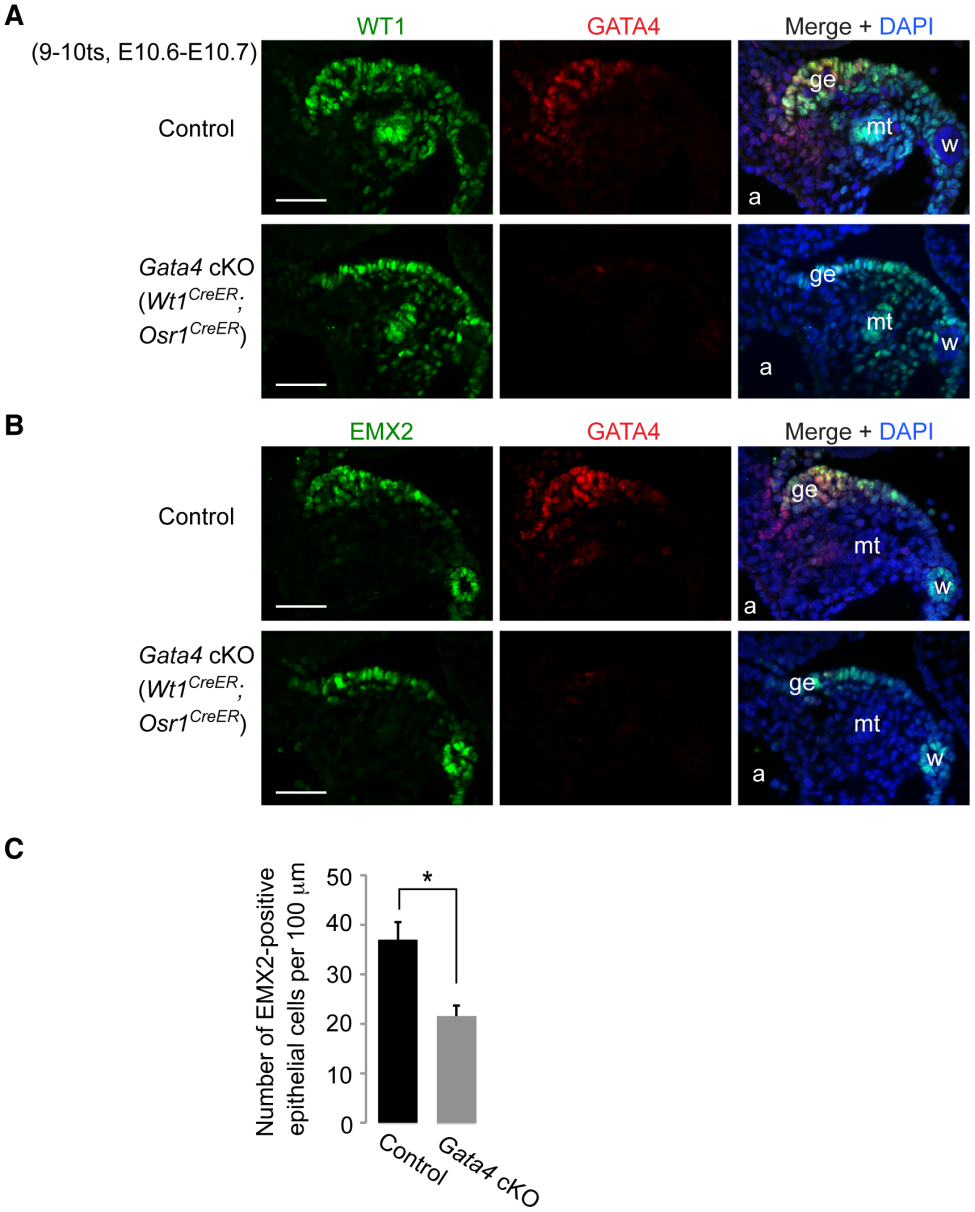
*Gata4* is not required for expression of WT1 and EMX2 in genital ridge epithelium. (A and B) Immunofluorescent staining for WT1, EMX2, and GATA4 protein in transverse sections of control and *Gata4* cKO (*Wt1^CreER^*;*Osr1^CreER^*) embryos. a, dorsal aorta; ge, genital ridge epithelium; mt, mesonephric tubule; w, Wolffian duct. Scale bars: 50 µm. (C) Comparison of numbers of EMX2-positive cells per 100 µm (along length of epithelial surface) in control and *Gata4* cKO (*Wt1^CreER^*;*Osr1^CreER^*) embryos. Cells were counted on transverse sections of embryos taken at similar A-P positions. Plotted here are means ± standard deviation from biological replicates (n = 3 for each genotype). *, *P* = 0.003 (two-tailed Student's *t*-test).

In contrast to the dependence of LHX9 and SF1 expression on *Gata4*, both WT1 and EMX2 were expressed in the coelomic epithelium of *Gata4* cKO (*Wt1^CreER^*;*Osr1^CreER^*) and *Gata4* cKO (*CAG-CreER*) embryos (Figure 5 and Figure S3). In addition, the overall expression patterns of WT1 and EMX2 in the mesonephric region were indistinguishable between control and *Gata4* cKO embryos. Therefore, WT1 and EMX2 are not downstream of GATA4. Although both *Wt1* and *Emx2* play an essential role in genital ridge development, they are not required to initiate the process 13,14. Likewise, *Gata4*, which is required for initiation, is present in both *Wt1*- and *Emx2*-deficient genital ridges [7], [38]. Thus, regulation of *Wt1* and *Emx2* is independent of *Gata4* in gonadogenesis, and vice versa.

The presence of EMX2 staining in the coelomic epithelium of *Gata4* cKO embryos allowed us to quantitate the reduction in epithelial cell number in *Gata4* cKO embryos, compared to controls. We counted the number of EMX2-positive coelomic epithelial cells per unit length. By E10.6–10.7, the EMX2-positive epithelial layer in *Gata4* cKO (*Wt1^CreER^*;*Osr1^CreER^*) embryos contained about half the number of cells seen in controls (Figure 5C), reflecting the absence of epithelial thickening in *Gata4* cKO embryos.

### *Gata4* deficiency does not interfere with primordial germ cell migration

**Figure 6:**
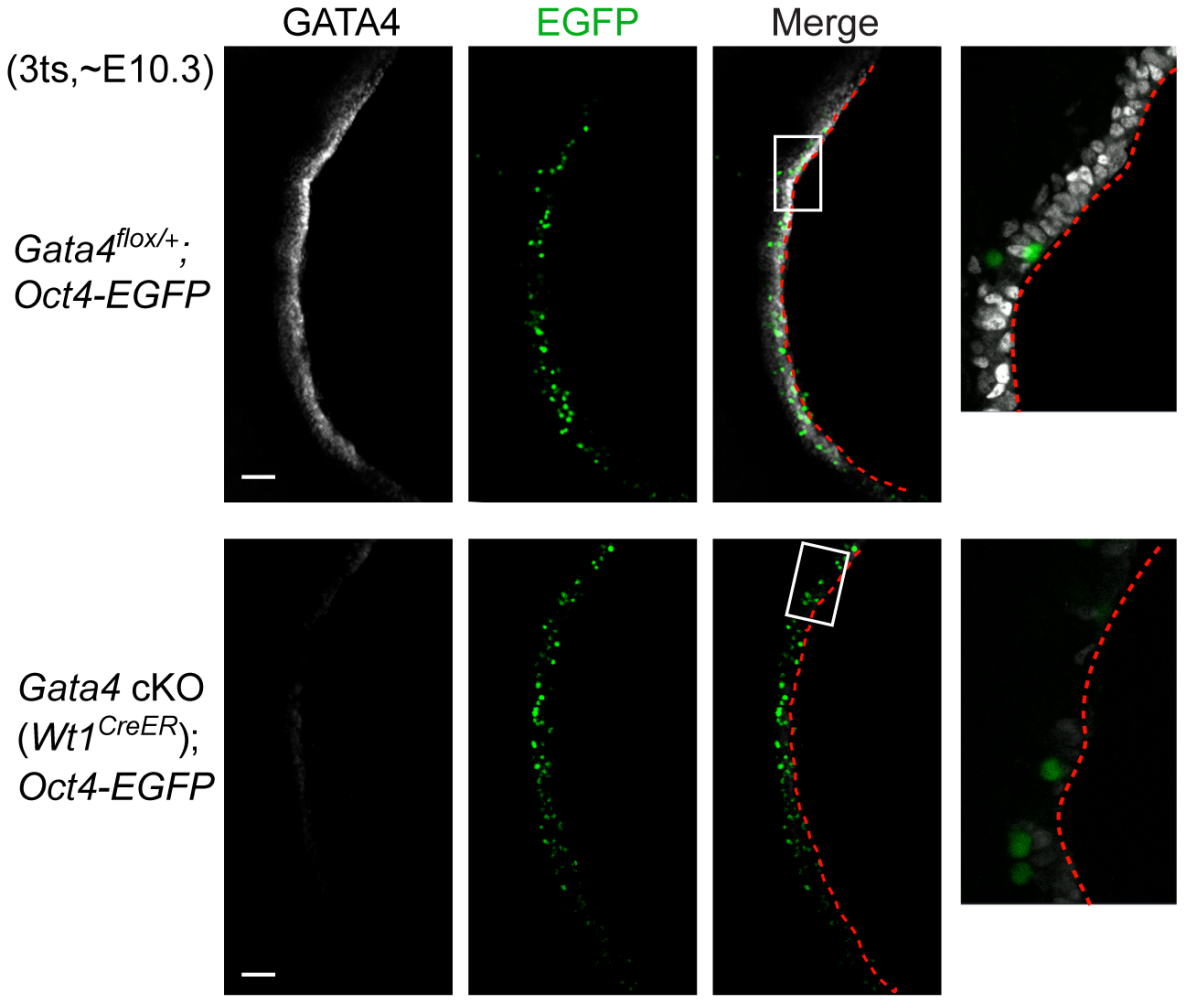
Migration of primordial germ cells is unaffected in *Gata4* cKO embryos. Whole-mount immunofluorescent staining for GATA4 protein (grey) in control and *Gata4* cKO (*Wt1^CreER^*) embryos. Confocal images were taken sagittally for a longitudinal view of the genital ridge. *Oct4-EGFP* transgene marks germ cells (green). Panels on far right provide higher magnification views of boxed areas. Red dashed lines outline the coelomic epithelial surface. Scale bars: 50 µm.

Primordial germ cells (PGCs) migrate from the base of the allantois to the developing genital ridge, arriving there between E10.0 and E11.5 [39]–[41]. A defect in genital ridge growth does not block PGC migration in *Lhx9−/−*, *Sf1−/−*, *Wt1−/−*, and *Emx2−/−* mutants [11]–[14]. One possible explanation is that the initial genital ridge formation seen in these mutants is sufficient to attract PGCs. Having shown that the genital ridge does not begin to form in the absence of *Gata4*, we tested whether migration of PGCs was affected in *Gata4* cKO embryos. We performed whole-mount immunofluorescence on embryos at E10.3 (3 ts), when PGCs are arriving at the genital ridges. In control embryos (*Gata4^flox/+^;Oct4-EGFP*), a longitudinal view showed that GATA4-expressing cells were present along the entire length of the genital ridge, and PGCs marked by an *Oct4-EGFP* transgene [42] had arrived in the coelomic epithelium of the genital ridge at this time point (Figure 6). In *Gata4* cKO (*Wt1^CreER^*);*Oct4-EGFP* embryos, GATA4 was absent yet PGCs still migrated to the corresponding coelomic epithelium despite the absence of genital ridge formation (Figure 6). We next looked at transverse sections of mesonephric regions from additional lines, *Gata4* cKO (*CAG-CreER*) and *Gata4* cKO (*Wt1^CreER^*;*Osr1^CreER^*). We found that PGCs, labeled with germ cell marker SSEA1, had migrated to the coelomic epithelium on the ventromedial side of the mesonephros despite the absence there of the epithelial thickening that would normally mark the beginning of genital ridge formation (Figure S4). These results suggest that neither *Gata4* nor gonadal development is required for migration of PGCs during embryogenesis.

## Discussion

**Figure 7:**
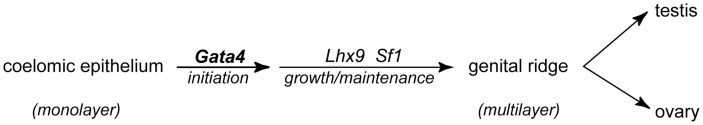
A proposed model for formation of the genital ridge. Formation of the testis or ovary begins when *Gata4*-dependent thickening of the coelomic epithelium gives rise to the genital ridge. LHX9 and SF1, acting downstream of GATA4, are subsequently induced, promoting growth and maintenance of the genital ridge. WT1 and EMX2 (not shown), though widely expressed in the genital ridge, are not dependent on *Gata4* and likely function in parallel to support growth and maintenance of the genital ridge. The genital ridge then develops as either an ovary or a testis, depending on the sex chromosome constitution of the embryo.

The genital ridge is the sexually undifferentiated, or bipotential, precursor of testis or ovary. We explored the question of how formation of the genital ridge is initiated. More specifically, we investigated the genetic regulation of the thickening of the coelomic epithelium, which constitutes the first step in gonadogenesis. Our data show that GATA4 expression in the coelomic epithelium precedes thickening and progresses in an A-P direction, correlating well with the A-P progression of genital ridge formation [1]. When we conditionally deleted *Gata4* from the E8.75 embryos, we found that formation of the genital ridge was *Gata4*-dependent. Our results were consistent using a number of different tamoxifen-inducible Cre drivers. Collectively, in the absence of *Gata4*, we did not observe the cellular changes critical for thickening of the coelomic epithelium, such as increasing proliferation and basement membrane fragmentation. Therefore, the coelomic epithelium shows no features of gonadal differentiation in the absence of *Gata4*. Failure of genital ridge formation is further confirmed by the lack of LHX9 and SF1 expression in *Gata4* cKO embryos. Thus, *Gata4* is the first genetic regulator shown to be required for the initiation of gonadogenesis (Figure 7).

*Gata4* has been previously studied as an important regulator of the testis determination pathway [27]–[30]. Correct dosage of fully functional *Gata4* is critical for properly initiating the testis differentiation program in the genital ridge. Manuylov *et al*. showed that when *Gata4* deletion was induced by tamoxifen injection at E10.5 via *Wt1^CreER^*, testis differentiation was blocked and sex reversal was observed [30]. When *Gata4* deletion was induced later, at E11.5, testis formation occurred although the testicular cords were somewhat disorganized [30]. In the present study, we induced *Gata4* deletion by tamoxifen injection at an earlier time point, E8.75, and noted the complete absence of genital ridge formation in both XX and XY embryos. These results suggest that *Gata4* plays at least two crucial roles in early gonadal development: initiation of genital ridge formation, followed by testis differentiation of the genital ridge. Therefore, the specific timing of *Gata4* loss leads to divergent phenotypes in mouse embryonic gonads. Moreover, previous studies of the *Gata4^ki^* allele have indicated the importance of GATA4-FOG interaction in mouse testis differentiation [28], [29]. Because the genital ridge is still formed in *Gata4^ki^* animals, GATA4 likely exerts its specific function in gonadal initiation independent of its interaction with FOG cofactors.

Our *Gata4* cKO embryos died before ∼E11.5, due to broad *Gata4* deletion induced by the *CreER* or *CreERt2* drivers used in the present study. This early death prevented us from studying sexual differentiation and later development of embryos that lack a gonad. We did not observe any signs of coelomic epithelial thickening, or expression there of LHX9 or SF1, in *Gata4* cKO embryos. However, we cannot rule out the possibility that the onset of gonadogenesis may simply be delayed in these mutants. Therefore, generation of a genital ridge-specific *Cre* mouse line will be necessary for future studies.

We have shown that *Lhx9* and *Sf1* are not expressed in the coelomic epithelium of *Gata4* cKO embryos, indicating that expression of these genes depends on *Gata4*. Given that the promoters of both genes contain consensus GATA4 binding sites [43], [44], it is plausible that GATA4 may directly activate *Lhx9* and *Sf1* transcription in the coelomic epithelium. However, GATA4 is known to function as both a transcriptional activator and a transcriptional repressor. For instance, in the epicardial mesothelium, GATA4, together with its cofactor FOG2, suppresses *Lhx9* expression at E11.5 by binding directly to conserved binding sites in the promoter [43]. Furthermore, an *in vitro* biochemical study suggests that GATA4 can either activate or inhibit the activity of the *Sf1* promoter through the consensus binding site depending on the cellular environment [44]. In the cellular context of the coelomic epithelium, it is possible that GATA4 directly stimulates the *Lhx9* and *Sf1* promoters by cooperating with a specific set of cofactors. Another possibility is that GATA4 indirectly activates transcription of *Lhx9* and *Sf1* through repression of their repressors.

The regulation of *Lhx9* expression is rather specific to *Gata4*, as *Lhx9* expression is affected in *Gata4* cKO but not in *Sf1−/−*, *Wt1−/−*, and *Emx2−/−* mutants [7], [12], [45]. In contrast, *Sf1* expression is inactivated in the coelomic epithelia of all known mutants defective in genital ridge development, including *Lhx9−/−*, *Wt1−/−*, *Emx2−/−*, and *Gata4* cKO embryos [7], [12], [45], implying that *Sf1* may be a common downstream target of multiple signaling cascades that control the formation and growth of the genital ridge. Indeed, the *Sf1* promoter contains functional binding sites for multiple factors, including GATA4, WT1, and LHX9 [44], [45]. We also found that expression of SF1 is restricted to the genital ridge epithelium, while expression of GATA4 and LHX9 extends to the dorsal end of the mesentery area (Figure 1D and Figure 4B, 4C) [7]. Therefore, these data imply that SF1 marks the identity of true gonadal somatic precursor cells.

GATA4 is a transcription factor whose functions appear to be conserved among vertebrates. For instance, *Gata4^−/−^* ES cell-derived mouse embryos and *Gata4*-deficient zebrafish embryos have strikingly similar phenotypes, including defects in heart tube looping, ventricle expansion, liver bud expansion, and derivation of the pancreas from the foregut [20], [46]–[48]. *Xenopus* embryos also require *Gata4* for heart and liver development [49]. Considering the conserved expression of *Gata4* in the genital ridge across vertebrates [21]–[26], we propose that the gonad is initiated through an evolutionarily conserved mechanism in which differentiation of the coelomic epithelium into the genital ridge is dependent on a common regulator, *Gata4*. In the current study, we established that *Lhx9* and *Sf1* act downstream of *Gata4* during the initiation of gonadogenesis in mouse embryos (Figure 4). Conserved expression of *Lhx9* and *Sf1* with *Gata4* in the genital ridges of different species would support a conserved role of *Gata4* in gonadal initiation. Indeed, *Gata4*, *Lhx9*, and *Sf1* are all expressed in the genital ridges, in both sexes, of chicken embryos [24]. *Sf1* was also detected in the genital ridges of dogs and turtles [50], [51].

PGCs migrate from the base of the allantois through the hindgut and mesentery to their final destination, the gonads, where they ultimately give rise to gametes. Our data show that PGCs were able to migrate to the coelomic epithelium on the ventromedial side of the mesonephros in *Gata4* cKO embryos (Figure 6 and Figure S4), suggesting that neither *Gata4* nor genital ridge formation is required for PGC migration. In agreement with this finding, it has been shown that PGCs actively migrate out of the hindgut toward the presumptive gonadal region at ∼E9.5 before the genital ridges are formed [41]. In addition, somatic factors that guide PGC migration, such as the chemokine SDF1/CXCL2 and the transcription factor FOXC1, are expressed not only in genital ridges, but also in the mesentery and mesonephros, starting as early as E9.0 [52]–[54]. We conclude that these somatic factors, expressed independently of *Gata4*, are sufficient to direct PGCs to their final destination, or to its proximity, even in the absence of the genital ridges.

*Gata4* cKO mutants show no signs of gonadal initiation or differentiation, unlike the previously reported mutants (*Lhx9−/−*, *Sf1−/−*, *Wt1−/−*, and *Emx2−/−*) where the genital ridge is formed but then degenerates. *Gata4* exhibits a functional role in gonadogenesis earlier than *Lhx9*, *Sf1*, *Wt1*, and *Emx2*. Notably, GATA4 is a transcription factor that lies in the middle of signaling cascades. Identification of upstream regulators and additional downstream targets of *Gata4* will provide insights into the regulation of gonadal initiation. Thus, our findings open to study the earliest steps in the formation of testes and ovaries.

## Materials and Methods

### Ethics statement

All experiments involving mice were approved by the Committee on Animal Care at the Massachusetts Institute of Technology.

### Mice

*Gata4^flox/+^* [47], *CAG-CreER* [32], *Osr1^eGFP-CreERt2/+^* [33], *Wt1^CreERt2/+^* [34], and *Gt(ROSA)26Sor^CAG-tdTomato^* mice were obtained from Jackson Laboratory (Stock Numbers 008194, 004682, 009061, 010912, and 007908, respectively) and then intercrossed for the experiment. Mice carrying an *Oct4-EGFP* transgene were also obtained from Jackson Laboratory (Stock Number 004654) and then backcrossed to the C57BL/6 strain (Taconic Farms) for at least 11 generations. *Gata4*-conditional-mutant embryos were generated by mating males carrying *Gata4^+/Δ^* and the indicated *CreER* to *Gata4^flox/flox^* females. Tamoxifen (Sigma) was dissolved in corn oil (Sigma) at a concentration of 20 mg/ml. Dams were injected intraperitoneally at 8.75 days postcoitum with a single shot of tamoxifen (4–5 mg/40 g body weight) to induce excision of the floxed *Gata4* allele. The injection scheme was optimized for maximum embryo survival and *Gata4* excision efficiency. Embryos were collected between E10.0 and E11.5; tail somites were counted to determine precise age. Genotypes were assayed by PCR according to protocols from the Jackson Laboratory website.

### Whole-mount immunofluorescent staining

Mouse embryos were either left whole or dissected to remove heads, limbs, body walls, and internal organs. Embryos were fixed at 4°C overnight in 4% paraformaldehyde and then blocked with 3% BSA/5% donkey serum/0.1% Triton X-100/PBS for another night. After washing with 0.1% Triton X-100/PBS, embryos were incubated at 4°C overnight with antibodies against GATA4 (sc-25310 or sc-1237, Santa Cruz Biotechnology), SF1 (PP-N1665-00, R&D Systems) and/or LHX9 (sc-19348, Santa Cruz Biotechnology). After washing for at least 8 hours, embryos were then incubated at 4°C overnight with donkey secondary antibodies conjugated with FITC, Rhodamine Red X, or DyLight 649 (Jackson ImmunoResearch). All antibodies were diluted 1∶100 in 1% BSA/0.1% Triton X-100/PBS solution. After washing for at least 8 hours, embryos were preserved in SlowFade Gold Antifade reagent (Life Technologies). Images were taken using an LSM710 confocal microscope (Zeiss). For transverse views, embryos were embedded in 7.5% low-melting point agarose, cut into 300 µm-thick transverse sections with a vibratome, and imaged via confocal microscopy.

### Immunohistochemistry/immunofluorescence

Immunohistochemical staining of embryonic sections was carried out as described previously [55]. Briefly, whole embryos were fixed at 4°C overnight in 4% paraformaldehyde, paraffin embedded, and sectioned. Sections representing the anterior portion of the genital ridges were used for all experiments. Slides were then dewaxed, rehydrated, and antigen-retrieved by microwaving in citrate buffer. After blocking, slides were incubated with primary antibodies. For colorimetric staining, slides were incubated with rabbit or mouse ImmPress reagent (Vector Labs), developed using DAB substrate kit (Vector Labs), and counterstained with hematoxylin. For fluorescent staining, slides were incubated with donkey secondary antibodies conjugated with FITC, Rhodamine Red X or DyLight 649 (Jackson ImmunoResearch) and mounted with ProLong Gold Antifade reagent with DAPI (Life Technologies).

For BrdU incorporation experiments, pregnant mice were injected intraperitoneally with BrdU (100 mg/kg body weight) six hours before sacrifice. Embryos were removed and processed for immunofluorescent staining, following the procedure described above, except that slides were denatured with 3.5N HCl for 30 seconds before blocking. Germ cells, characterized by their large, round, clear nuclei with clumps of chromatin scattered around the nuclear periphery, were excluded from counting. TUNEL staining was carried out with In Situ Cell Death Detection Kit (Roche Applied Science) according to the manufacturer's instructions.

Primary antibodies against GATA4 (sc-25310, Santa Cruz Biotechnology), SF1 (PP-N1665-00, R&D Systems), laminin (L9393, Sigma), BrdU (OBT0030, Accurate Chemical and Scientific), WT1 (RB-9267, Thermo Scientific), and EMX2 (a kind gift of Ken-ichirou Morohashi, Kyushu University, Fukuoka, Japan) [7], [56] were used in the study.

### Quantitative RT-PCR

Urogenital ridges were collected, submerged in TRIzol (Life Technologies), and then stored at −80°C until genotyping was completed. Total RNA was prepared according to the manufacturer's instructions and DNase-treated using DNA Free Turbo (Ambion). Three hundred ng of total RNA was reverse transcribed, and qPCR was performed with SYBR Green dye, as previously described [55]. qPCR primer pairs employed were as follows:

5′- CGGAAGCCCAAGAACCTGAATAAATC-3′ and

5′- GCTGCTGTGCCCATAGTGAGATGAC-3′ for *Gata4*,

5′- GAGTTCGTCTGTCTCAAGTTCCTCATCC-3′ and

5′- ACCTCCACCAGGCACAATAGCAAC-3′ for *Sf1*,

5′- ACCAGCAGCCTTATCCACCTTCACAG-3′ and

5′- TGTAATGCCCCAAGATTTGTTCTCCC-3′ for *Lhx9*, and

5′- GAGAGCCAGCCTACCATCC-3′ and

5′- GGGTCCTCGTGTTTGAAGGAA-3′ for *Wt1*.

Results were analyzed using the delta-delta Ct method with *β-actin* as a normalization control.

## Supporting Information

Figure S1 GATA4 expression is restricted to the genital ridge. Whole-mount immunofluorescent staining for GATA4 (green) and PAX2 (red, antibody from Abcam, ab79389) protein in wild-type embryos at the 6 tail-somite stage (∼E10.4). Confocal images of urogenital ridges were taken sagittally. PAX2 marks the Wolffian duct. Border between mesonephric and metanephric regions is defined by location of 27–28^th^ somites of the embryo. Scale bars: 200 µm.
(TIF)

Figure S2 *Gata4* is required for expression of SF1 in genital ridge epithelium. IHC staining for SF1 protein (brown) in sections of control and *Gata4* cKO (*CAG-CreER*) embryos. Arrows indicate examples of positive SF1 staining. a, dorsal aorta; m, mesentery; mt, mesonephric tubule; w, Wolffian duct. Scale bars: 50 µm.
(TIF)

Figure S3 *Gata4* is not required for expression of WT1 and EMX2 in genital ridge epithelium. IHC staining for WT1 and EMX2 protein (brown) in transverse sections of control and *Gata4* cKO (*CAG-CreER*) embryos. Sections were chosen to represent similar A-P positions in embryos. a, dorsal aorta; ge, genital ridge epithelium; mt, mesonephric tubule; w, Wolffian duct. Scale bars: 50 µm.
(TIF)

Figure S4 Primordial germ cells migrate to the coelomic epithelium on the ventromedial side of the mesonephros in *Gata4* cKO embryos. (A and B) Immunofluorescent staining for GATA4 (red) and SSEA1 (green, antibody from Chemicon, MAB4301) protein in sections of control and *Gata4* cKO embryos. SSEA1 marks germ cells, which are indicated by arrows. Nuclei stained with DAPI (blue). Yellow dashed lines outline the coelomic epithelial surface. a, dorsal aorta; m, mesentery; mt, mesonephric tubule. Scale bars: 50 µm.
(TIF)
