# Supplementary figures and images for: Gata4 Is Required for Formation of the Genital Ridge in Mice
Source: PLoS Genet. 2013 Jul 11;9(7):e1003629. doi: 10.1371/journal.pgen.1003629 (PMC3708810; doi:10.1371/journal.pgen.1003629)

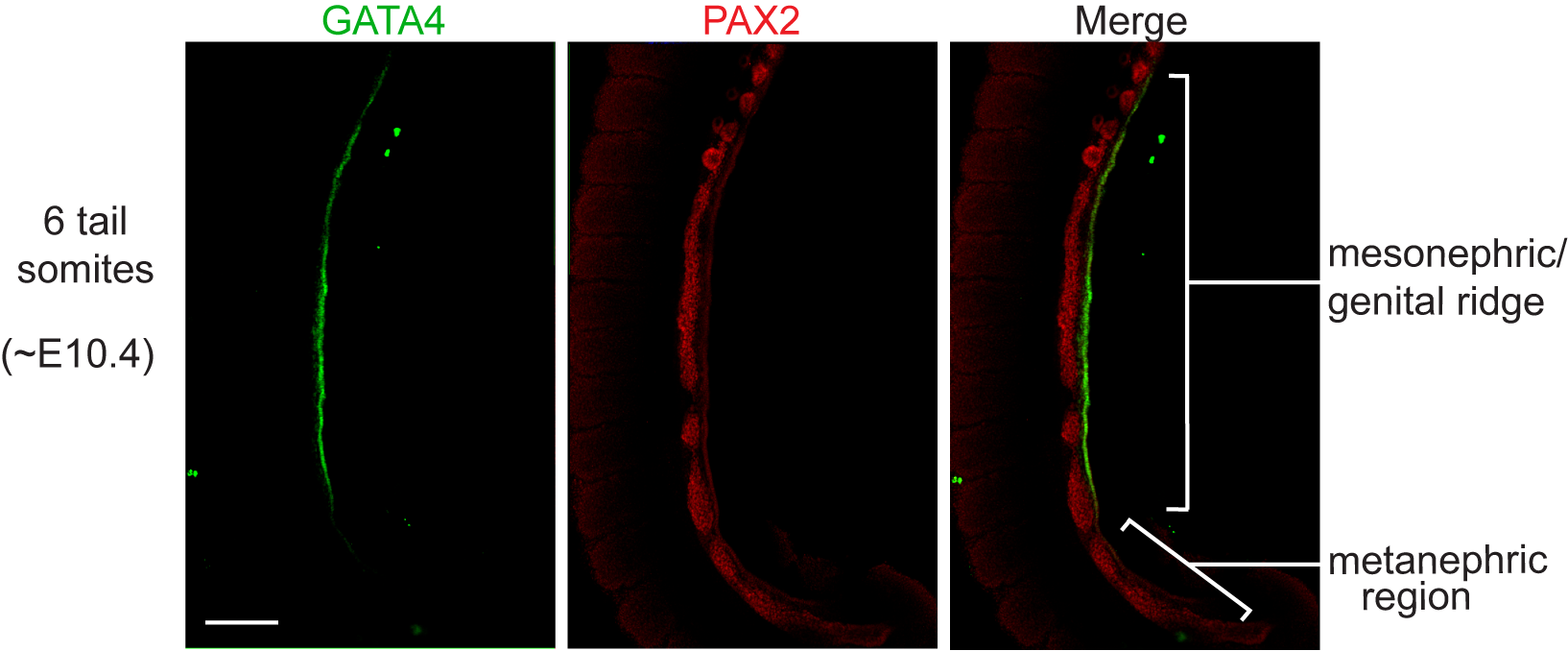

Supplement: Figure S1 — GATA4 expression is restricted to the genital ridge. Whole-mount immunofluorescent staining for GATA4 (green) and PAX2 (red, antibody from Abcam, ab79389) protein in wild-type embryos at the 6 tail-somite stage (∼E10.4). Confocal images of urogenital ridges were taken sagittally. PAX2 marks the Wolffian duct. Border between mesonephric and metanephric regions is defined by location of 27–28th somites of the embryo. Scale bars: 200 µm. (TIF) [file pgen.1003629.s001.tif]

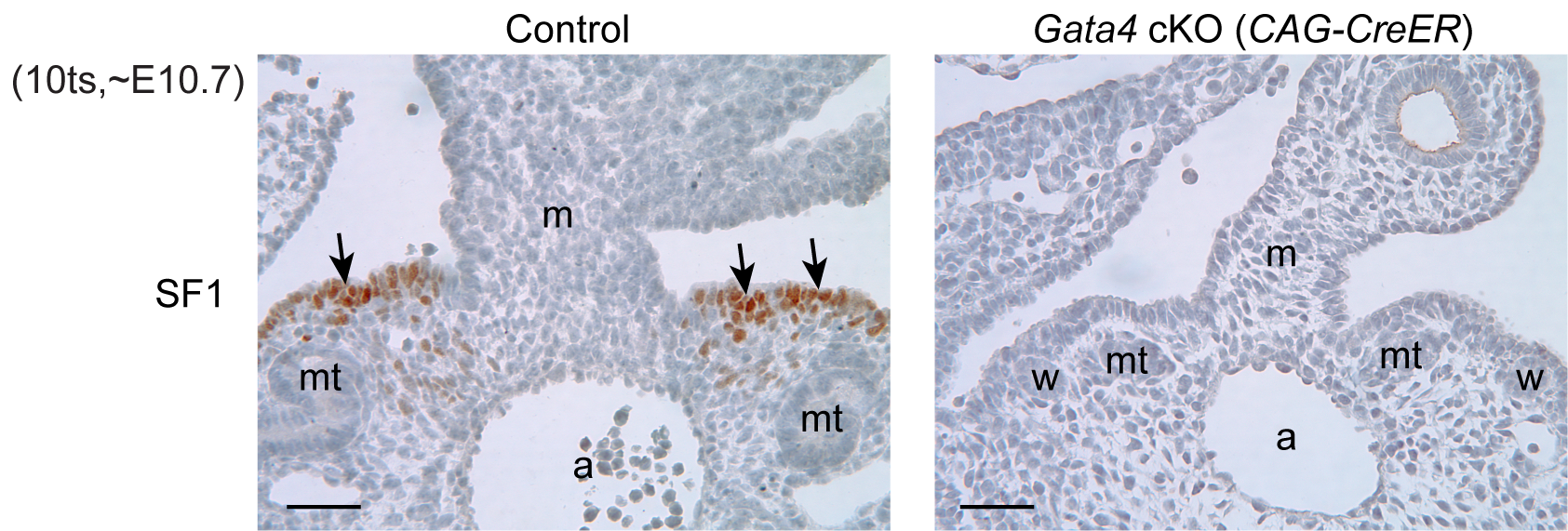

Supplement: Figure S2 — Gata4 is required for expression of SF1 in genital ridge epithelium. IHC staining for SF1 protein (brown) in sections of control and Gata4 cKO (CAG-CreER) embryos. Arrows indicate examples of positive SF1 staining. a, dorsal aorta; m, mesentery; mt, mesonephric tubule; w, Wolffian duct. Scale bars: 50 µm. (TIF) [file pgen.1003629.s002.tif]

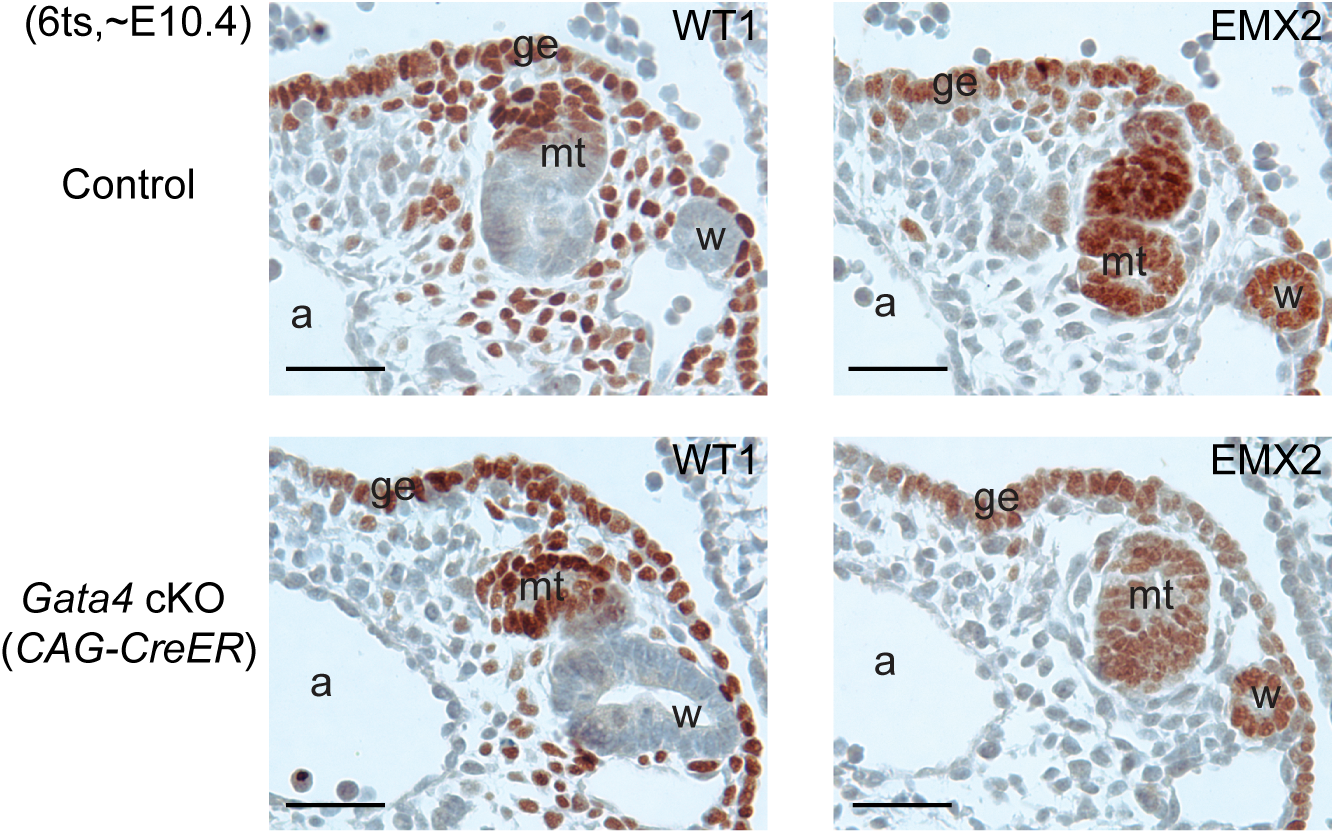

Supplement: Figure S3 — Gata4 is not required for expression of WT1 and EMX2 in genital ridge epithelium. IHC staining for WT1 and EMX2 protein (brown) in transverse sections of control and Gata4 cKO (CAG-CreER) embryos. Sections were chosen to represent similar A-P positions in embryos. a, dorsal aorta; ge, genital ridge epithelium; mt, mesonephric tubule; w, Wolffian duct. Scale bars: 50 µm. (TIF) [file pgen.1003629.s003.tif]

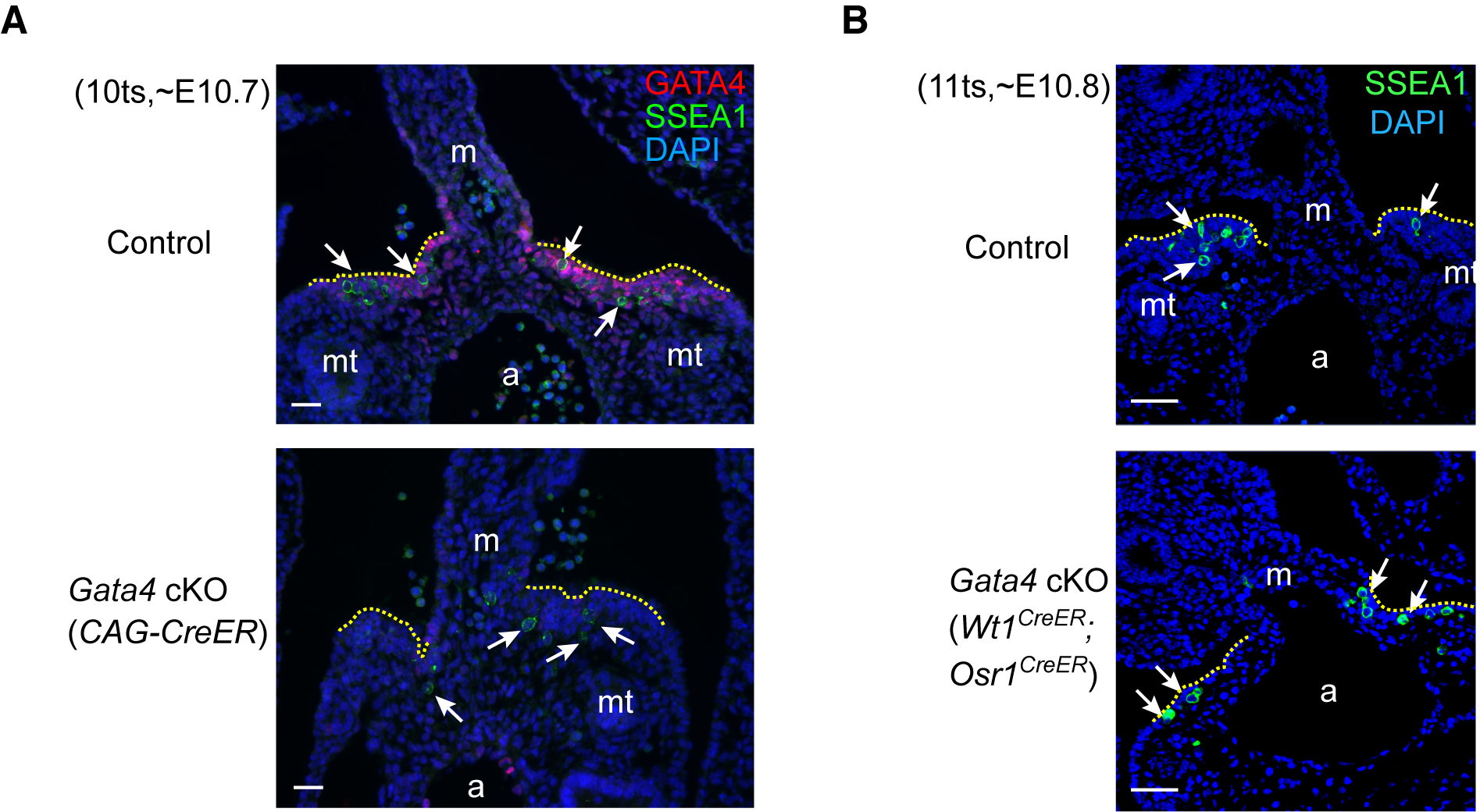

Supplement: Figure S4 — Primordial germ cells migrate to the coelomic epithelium on the ventromedial side of the mesonephros in Gata4 cKO embryos. (A and B) Immunofluorescent staining for GATA4 (red) and SSEA1 (green, antibody from Chemicon, MAB4301) protein in sections of control and Gata4 cKO embryos. SSEA1 marks germ cells, which are indicated by arrows. Nuclei stained with DAPI (blue). Yellow dashed lines outline the coelomic epithelial surface. a, dorsal aorta; m, mesentery; mt, mesonephric tubule. Scale bars: 50 µm. (TIF) [file pgen.1003629.s004.tif]
